# Nitrous Oxid Gas, Essence of Orange, Ether and Sequestration in General Anesthesia for Operation in the Upright Position

**Published:** 1913-09

**Authors:** Thomas R. French

**Affiliations:** Brooklyn


### Nitrous Oxid Gas, Essence of Orange, Ether and Sequestration in General Anesthesia for Operation in the Upright Position.

We are now able to state that as a result of the movements of the new operating table, and also as a result of the tests made in our clinic at the Long Island Hospital, that operative work in the upright position can be done with still less loss of blood, with the need of still less of the anesthetic, and with less disagreeable conditions during recovery from the anesthetic. Sudden changes in posture or sudden disturbances of the body while the patient is under a general anesthetic, predisposes to shock, the effects of which are manifested both during the operation and during the recovery from anesthesia. After a careful study of this phenomenon, I became impressed with the value of elevation of the body from the recumbent position to the upright position without jarring, and this led to the construction of the chair table. By the aid of a new attachment all the movements of the body are now made with as great freedom from jarring and disturbance of balance, with infants and very small children, as had hitherto been possible only with adults or larger children. The resulting improvement in conditions leaves no doubt of the accuracy of the observation. One of the most important and valuable recent additions to methods of anesthesia is the ability to omit or bridge the second stage, or stage of excitement, and we have been impressed with the desirability of attaining narcosis without struggle. This can be accomplished with nitrous oxid gas, but with greater ease and certainty with the essence of orange. The discoverer of the remarkable effects of the essence of orange as a preliminary to ether, Dr. Gwathmey, of New York, has demonstrated the possibilities and has proven to our satisfaction that it greatly assists in the reduction of shock by bridging the stage of excitement. Anesthesia conducted in this way up to the time of raising the body to the upright position is a contributory factor in reducing hemorrhage and also in reducing the quantity of anesthetic and in shortening and modifying the anesthetic after-effects. Irrespective of all other conditions, there is a well-defined relationship between the degree of skill in which a patient is anesthetized in the upright position and the amount of hemorrhage is reduced if the anesthetic is smoothly given. A preliminary use of nitrous oxid gas or the essence of orange and the drop method of anesthol, followed by the drop method of ether with the open inhaler, and later with the Allis inhaler, will insure a prolongation of the anesthesia after the body has reached the upright position, a reduction of hemorrhage, and a reduction in disagreeable sequelæ. Sequestration of the limbs produces a congestive hyperemia, and is produced by means of inflated blood pressure cuffs about the thighs and arms; this reduces the amount of blood in the head. This, in association with the upright position which has been carried out in fifty-eight cases during the past season, reduces still further the amount of anesthetic required and still further the loss of blood. There need be no apprehension of subsequent bleeding.—Dr. Thomas R. French, Brooklyn.

